# Isolation, cultivation, and application of primary respiratory epithelial cells obtained by nasal brushing, polyp samples, or lung explants

**DOI:** 10.1016/j.xpro.2022.101419

**Published:** 2022-05-27

**Authors:** Anita Golec, Iwona Pranke, Paolo Scudieri, Kate Hayes, Elise Dreano, Fiona Dunlevy, Aurelie Hatton, Damian G. Downey, Luis Galietta, Isabelle Sermet

**Affiliations:** 1 Institut Necker Enfants Malades, Faculté de Médecine Necker, INSERM U1151, Université de Paris, 160 rue de Vaugirard, 75015 Paris, France; 2 Medical Genetics Unit, Istituto Giannina Gaslini, 16147 Genova, Italy; 3 Department of Neurosciences, Rehabilitation, Ophthalmology, Genetics, Maternal and Child Health (DiNOGMI), University of Genova, 16147 Genova, Italy; 4 Northern Ireland Clinical Research Facility, The Wellcome-Wolfson Institute of Experimental Medicine, Queen’s University, U Floor, City Hospital, Lisburn Road, Belfast, Northern Ireland; 5 European Cystic Fibrosis Society, Karup, Denmark; 6 The Wellcome-Wolfson Institute of Experimental Medicine, Queen’s University, Lisburn Road, Belfast, Northern Ireland; 7 Telethon Institute of Genetics and Medicine (TIGEM), 80078 Pozzuoli, Napoli, Italy; 8 Department of Translational Medical Sciences, Università di Napoli “Federico II,” 80131 Napoli, Italy; 9 Service de Pneumologie et Allergologie Pédiatriques, Centre de Ressourcés et de Compétence de la Mucoviscidose, Hôpital Necker Enfants Malades, 149 rue de Sévres, Paris, France; 10 INSERM U1151, Institut Necker Enfants Malades, Université de Paris, 160 rue de Vaugirard, 75015 Paris, France

**Keywords:** Cell Biology, Cell culture, Cell isolation, Clinical Protocol

## Abstract

Here, we present a standardized protocol for isolation, maintenance, and polarization of the respiratory epithelial primary cells from patient samples acquired from nasal brushing, polyp specimens, or lung explants. This protocol generates a clearly defined polarized layer of epithelial cells on filters, with a good number of ciliated cells and a thin layer of mucus. We detail the steps for samples prepared from patients with cystic fibrosis as well as from subjects without cystic fibrosis.

## Highlights

• Protocol for isolating and maintaining respiratory epithelial cells from patient samples
• Generates polarized cells with ion transport properties on filters
• Can be used for electrophysiological measurements using an Ussing system
• Applicable for airway surface liquid volume analysis or immunofluorescent staining

## Abstract (editor highlights)

Publisher’s note: Undertaking any experimental protocol requires adherence to local institutional guidelines for laboratory safety and ethics.

## Graphical abstract

**Figure:**
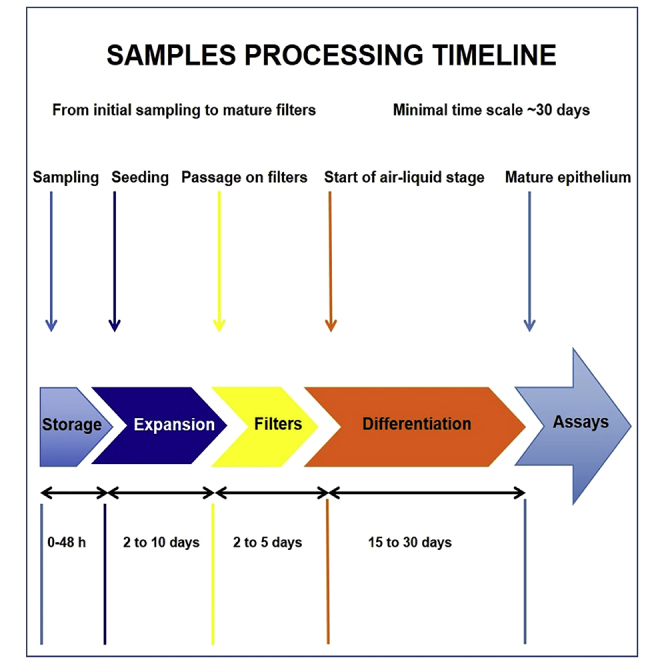

## Before you begin

This protocol presents all the necessary information for conducting successful cell culture without co-culture of the feeder cells (fibroblasts). Non-proliferating, yet metabolically active fibroblasts can be used to boost cultures that originate from very low starting cell inoculum. Feeder cells support improved attachment of the desired cells and secrete growth factors and nutrients into the culture medium. The mitotic arrest of the feeder cells is often achieved by either Mitomycin C treatment or gamma-ray irradiation. Both of these methods confer a hazardous risk to the operator. Mitomycin C is regarded as a probable human carcinogen and the availability of gamma irradiation sources is very limited. Therefore, we have decided to omit the use of the feeder cells in this protocol. We acknowledge the potential beneficial effects of the co-culture and do not exclude the possibility of other laboratories conducting these cultures.

We have included procedures for 1) nasal brushing, and handling of polyp and lung explant samples; and 2) freezing/defrosting for biobanking of samples involved in clinical trials.

Please note, that despite strict adherence to the following protocol, a 100% success rate of the cell culture cannot be guaranteed, particularly for samples obtained by nasal brushing. The quality and quantity of the brushed material, variability in patients’ characteristics (including concomitant infections), as well as the specific CF mutation, can all play a crucial role in obtaining a good quality culture. Limitations of this protocol may include variation from laboratory to laboratory in terms of the use of antibiotics. This protocol represents the authors’ preferred methods of choice and any variation in local site procedure should be discussed on a case-by-case basis. Similarly, there may be site-to-site variation in terms of use of different media for amplification.

For example, use of a “home-made” serum-free medium.

Important notes to consider before starting:

1. All procedures must be performed in sterile conditions to protect the staff from potential biological risks (e.g., sample-based pathogens); personal protective equipment must be worn as local site policy dictates.
2. A dedicated incubator should be allocated specifically to primary cell culture only at 37°C with 5% CO_2_.
3. Collagen coated filters and flasks should be prepared in advance (see materials and equipment).
4. Prepare media and buffers in advance (see materials and equipment).

SARS-CoV-2:

All personnel must adhere to local site guidelines/strategies to ensure maximum protection for the operator and participants. Please refer to your local institutional guidelines for conduct of studies/clinical procedures involving collection and analysis of samples. This may necessitate PCR testing of the nasal brushing sample to exclude contamination by the virus, please refer to your institutional guidelines.

This protocol has been approved by the ECFS CTN Standardization Committee.

### List of abbreviations

ALI: air-liquid interface.

CF: cystic fibrosis.

DMEM: Dulbecco’s Modified Eagle Medium.

DMEM/F12: Dulbecco’s Modified Eagle’s Medium/Nutrient Mixture F-12 Ham.

DMSO: Dimethyl sulfoxide.

DTT: 1.4 Dithiothreitol.

EGF: Epidermal growth factor.

FBS: Fetal bovine serum.

Ham’s/F12: Ham’s Nutrient Mixture F12.

HEPES: (4-(2-hydroxyethyl)-1-piperazineethanesulfonic acid) is a zwitterionic organic chemical buffering agent.

PBS or DPBS: Phosphate Buffered Saline or Dulbecco’s Phosphate Buffered Saline.

PBS-DTT-FTVC: Phosphate Buffered Saline-1.4 Dithiothreitol-Fungizone-Tazocillin-Vancomycin-Colomycin.

Peni/Strepto: Penicillin-Streptomycin.

PPE: personal protective equipment.

Ultroser G™: Serum Substitute.

WT: wild type, an individual having the normal phenotype; that is, the phenotype commonly found in a natural population of organisms.

Y -27632: inhibitor of Rho-associated protein kinases.

## Key resources table

**Table:** 

REAGENT or RESOURCE	SOURCE	IDENTIFIER
**Biological samples**		

Nasal cells from nasal brushing (from human subjects age range will be dependent upon the clinical trial protocol/clinical requirements, male or female)	Site specific	n/a
Nasal polyp (from human subjects age range will be dependent upon the clinical trial protocol/clinical requirements, male or female)	Site specific	n/a
Lung explants (from human subjects age range will be dependent upon the clinical trial protocol/clinical requirements, male or female)	Site specific	n/a

**Chemicals, peptides, and recombinant proteins**		

Colomycin	Sanofi	provided by Necker Hospital Pharmacy
Ciprofloxacin	Mylan	provided by Necker Hospital Pharmacy
Vancomycin	Mylan	provided by Necker Hospital Pharmacy
Xylocaine naphazoline 5% (Lidocaine) https://www.vidal.fr/medicaments/xylocaine-naphazoline-5-sol-p-appl-loc-17949.html	Vidal	3400955164357
Collagen type IV https://www.sigmaaldrich.com/GB/en/search/c7521?focus=products&page=1&perPage=30&sort=relevance&term=C7521&type=product	Sigma-Aldrich Merck	C-7521
DMEM: Dulbecco’s Modified Eagle Medium https://www.thermofisher.com/order/catalog/product/61965026?SID=srch-srp-61965026	Life Technologies	61965026
DMEM/F12: Dulbecco’s Modified Eagle’s https://www.thermofisher.com/order/catalog/product/12634010?SID=srch-hj-12634010	Life Technologies	12634010
Ham’s/F12: Ham’s Nutrient Mixture F12 https://www.thermofisher.com/order/catalog/product/11765054?SID=srch-srp-11765054	Life Technologies	11765054
DMSO: Dimethyl sulfoxide https://www.sigmaaldrich.com/GB/en/search/d2650?focus=products&page=1&perPage=30&sort=relevance&term=D2650&type=product	Sigma-Aldrich Merck	D2650
DTT: 1.4 Dithiothreitol https://www.sigmaaldrich.com/GB/en/search/d0632-25g?focus=products&page=1&perPage=30&sort=relevance&term=D0632-25g&type=product	Sigma-Aldrich Merck	D0632-25g
EGF: Epidermal growth factor https://www.thermofisher.com/antibody/product/Human-EGF-Recombinant-Protein/PHG0311	Life Technologies	PHG0311
Epinephrine https://www.sigmaaldrich.com/GB/en/search/e4375?focus=products&page=1&perPage=30&sort=relevance&term=E4375&type=product	Sigma-Aldrich Merck	E4375
FBS: Fetal bovine serum https://www.thermofisher.com/order/catalog/product/10270106?SID=srch-srp-10270106	Life Technologies	10270106
Fungizone (Amphotericin B) https://www.thermofisher.com/order/catalog/product/15290026?SID=srch-hj-15290026	Life Technologies	15290026
HEPES: (4-(2-hydroxyethyl)-1-piperazineethanesulfonic acid) https://www.sigmaaldrich.com/GB/en/search/h3375?focus=products&page=1&perPage=30&sort=relevance&term=H3375&type=product	Sigma-Aldrich Merck	H3375
Hydrocortisone https://www.sigmaaldrich.com/GB/en/search/hydrocortisone?focus=products&page=1&perPage=30&sort=relevance&term=Hydrocortisone&type=product	Sigma-Aldrich Merck	H6909
Insulin https://www.sigmaaldrich.com/GB/en/search/i0516?focus=products&page=1&perPage=30&sort=relevance&term=I0516&type=product	Sigma-Aldrich Merck	I0516
PBS or DPBS: Phosphate Buffered Saline or Dulbecco’s https://www.thermofisher.com/order/catalog/product/14190094?SID=srch-hj-14190094	Life Technologies	14190094
Peni/Strepto: Penicillin-Streptomycin https://www.thermofisher.com/order/catalog/product/15140130?SID=srch-hj-15140130	Life Technologies	15140130
Pronase https://www.sigmaaldrich.com/GB/en/search/10165921001?focus=products&page=1&perPage=30&sort=relevance&term=10165921001&type=product	Merck	10165921001
Tazocillin	Mylan	provided by Necker Hospital Pharmacy
Trypsin 0.25% https://www.thermofisher.com/order/catalog/product/25200056?SID=srch-hj-25200056	Life Technologies	25200056
Ultroser G™: Serum Substitute https://www.sartorius.com/en/products/cell-culture-media-buffers/general-media/serum-substitute	Sartorius	15950-017
Y-27632 https://www.selleck.eu/products/Y-27632-2hcl.html?gclid=CjwKCAjwoP6LBhBlEiwAvCcthO-wa3uFU_ZtYDcedex2rrEuJ00a2CoCbWDQ3UQlRM7t6QCw8ktiqxoCk5UQAvD_BwE	Selleckchem	S1049
Glacial acetic acid https://www.sigmaaldrich.com/GB/en/product/sigald/a6283	Sigma-Aldrich Merck	A6283
FertiCult™ https://fertipro.com/2019/12/10/ferticult-flushing-medium/	FertiPro NV	FLUSH020
Transwell Permeable Supports, 6.5 mm Inserts, 24 well plate, 0.4 μm Polyester Membrane	Corning Incorporated	3470

**Other**		

Sterile Politzer tweezers	Site specific	n/a
Sterile nose speculum	Site specific	n/a
Cotton wool (to make meshes)	Site specific	n/a
Clar mirror – head mirror (optionally)	Site specific	n/a
Gyneas® Endocervical Brush https://www.gyneas.com/brosse-sterile-diam-3-5mm-50.html	Gyneas	39368
Olympus® bronchial brush, diameter 2 mm, for 1.2 mm scope channel https://medical.olympusamerica.com/products/brushes/cytology-brushes	Olympus	BC-202D-1210/BC-202D-2010/BC-202D-3010/BC-202D-5010/BC-203D-2006
**EVOM2 (manual epithelial volt/ohm meter)** https://www.wpiinc.com/media/wysiwyg/pdf/EVOM2_IM.pdf (the meter models may differ).	World Precision Instruments	EVOM2

## Materials and equipment

Nasal Brushing Protocol Materials:

Kit containing materials required for sampling of one subject:

• Alcohol for disinfection.
• Paper tissues.
• Non-sterile gloves.
• Xylocaine naphazoline 5% (Lidocaine).
• Cotton to make meshes.
• Sterile Politzer tweezers previously disinfected.
• A pair of scissors.
• Sterile nose speculum previously disinfected (optionally).
• Clar mirror – head mirror (optionally).
• 2 × 15 mL tubes (tube A) with 2.5 mL of DPBS.
• For subjects under 5 years: Olympus® bronchial brush, diameter 2 mm, for 1.2 mm scope channel.
• For subjects over 5 years old: Gyneas® Endocervical Brush (Brushes will remain sterile if unopened).
• Appropriate PPE as required.

### Media preparation (15 min)

***Note:*** Antibiotics listed here are prescribed as standard antibiotics for the CF patients with active infection, if the antibiogram is known; it is advisable to add any additional specific antibiotic to the culture medium.

Antibiotics are obtained from the local site hospital pharmacy, thus sterile liquids for injections are added directly to the culture medium, powders are weighed and dissolved and filter-sterilized.

***Note:*** there may be variability from one laboratory to another concerning the medium used for expansion. This SOP provides guidance for one method; please refer to your local site and laboratory standards.

#### Transport medium

The lung explant or polyp should be put in a sterile 50 mL container with 40 mL of DMEM medium or PBS. If the sample will be stored for longer than 24 h, it is advisable to use DMEM/F12 medium that is more nutrient-rich. It is optimal to leave a few pre-prepared containers on the surgical ward.

#### Dissociation media

The same dissociation media is used in the preparation of the lung explants and polyp samples:

**Table:** PBS-DTT-FTVC (Prepare it on the day)

	Final concentration in the medium	Amount
DPBS × 1	–	**48 mL**
DTT Dithiothreitol	5 mM∗	37.8 mg
Fungizone	2.5 μg/mL	0.5 mL
Tazocillin	10 μg/mL	0.5 mL
Vancomycin	100 μg/mL	0.5 mL
Colomycin	16 μg/mL	0.5 mL
**Final volume**		**50 mL**

∗the DTT is added as a powder here and the amount is relatively small, the quantity to be dissolved is calculated for 50 mL of liquid.

**Table:** PBS-FTVC (Prepare it on the day)

	Final concentration in the medium	Amount
**DPBS × 1**	–	**48 mL**
Fungizone	2.5 μg/mL	0.5 mL
Tazocillin	10 μg/mL	0.5 mL
Vancomycin	100 μg/mL	0.5 mL
Colomycin	16 μg/mL	0.5 mL
**Final volume**		**50 mL**

**Table:** DMEM/F12-FTVC (Prepare it on the day)

	Final concentration in the medium	Amount
**DMEM/F12**	–	**48 mL**
Fungizone	2.5 μg/mL	0.5 mL
Tazocillin	10 μg/mL	0.5 mL
Vancomycin	100 μg/mL	0.5 mL
Colomycin	16 μg/mL	0.5 mL
**Final volume**		**50 mL**

**Table:** DMEM/F12-FBS20%-FTVC (Prepare it on the day)

	Final concentration in the medium	Amount
DMEM/F12	–	**38 mL**
Fungizone	2.5 μg/mL	0.5 mL
Tazocillin	10 μg/mL	0.5 mL
Vancomycin	100 μg/mL	0.5 mL
Colomycin	16 μg/mL	0.5 mL
FBS		10 mL
Final volume		**50 mL**

#### Culture media

The culture medium used is common for the cells originating from: nasal brushing, lung explants or polyps.

***Amplification medium*** (Shelf life of the ready media is estimated for around 4–8 weeks at 4°C).

**Table:** 

	Storage conditions	Amount	Final concentration in the medium
DMEM F12	4°C	86 mL	86%
FBS	4°C	10 mL	10%
Peni/Strepto	4°C	1 mL	100 U/mL
Tazocillin	4°C	1 mL	10 μg/mL
Fungizone	4°C	1 mL	2.5 μg/mL
Colomycin	4°C	1 mL	16 μg/mL
Ciprofloxacin	4°C	150 μL	3 μg/mL
Hydrocortisone	−20°C	400 μL	0.2 μM
Insulin	4°C	50 μL	5 μg/mL
Epinephrine	−20°C	100 μL	0.5 μg/mL
EGF (stock: 20 μg/mL)	−20°C	50 μL	10 ng/mL
Y-27632 (stock: 20 mM)	−80°C	50 μL	10 μM
Final volume		**100 mL∗**	

∗the bulk ingredients give a total 100 mL.

***Air-Liquid Interface medium (ALI)*** (shelf life of the ready media is estimated for around 4–8 weeks at 4°C).

If Ciprofloxacin is needed during the cell culture, it has to be withdrawn from the medium at least 5 days before planned assay.

**Table:** 

	Storage	Amount	Final concentration in the medium
DMEM F12	4°C	94 mL	94%
UltroserG	4°C	2 mL	2%
Peni/Strepto	4°C	1 mL	100 U/mL
Tazocillin	4°C	1 mL	10 μg/mL
Fungizone	4°C	1 mL	2.5 μg/mL
Colomycin	4°C	1 mL	16 μg/mL
Final volume		**100 mL**	

***Freezing medium*** (Shelf life of the ready media is estimated for around 4–8 weeks at 4°C, in any case if stored, the vial should be wrapped in aluminum foil to protect from light.)

**Table:** 

	Amount for 50 mL	Amount for 10 mL
F12 Nutrient Mixture	38.5 mL	7.7 mL
HEPES	1.5 mL	0.3 mL
FBS	5 mL	1 mL
DMSO	5 mL	1 mL
Final volume	**50 mL**	**10 mL**

### Aliquots

#### Enzymes

Trypsin.

Material needed:

• 100 mL bottle of trypsin is kept at −20°C.
• 15 mL sterile tubes.

Procedure:

• Defrost the trypsin bottle in the fridge (can be done overnight).
• Divide the trypsin between the 15 mL tubes in the quantity of 10 mL.
• Note on each tube the date and the content, freeze at −20°C and keep for up to 6 months.

Pronase.

Material needed:

• Powder is conserved at −20°C.
• Sterile double distilled (dd) water.
• Sterile 15 mL tubes.
• Vortex.
• Syringe filter.

Procedure:

• Dilute the Pronase powder in the sterile dd water in the concentration of 1 mg/mL.
• Vortex.
• Sterile filter.
• Prepare the 4 mL aliquots in the 15 mL tubes.
• Note on each tube the date and the content, freeze at −20°C and keep for up to 6 months.

#### Serum, growth factors and supplements

Ultroser G (2%).

Material needed:

• Powder stored at 4°C.
• Sterile dd water.
• Vortex.
• Sterile 15 mL tubes.

Procedure:

• Add 20 mL of dd water to the bottle containing Ultroser G.
• Mix very well, vortex for a short time.
• As mixing is creating the foam, leave the solution for around 60 min.
• Prepare the aliquots of 5 mL in the 15 mL tubes.
• Note on each tube the date and the content, freeze at −20°C and keep for up to 6 months.

Hydrocortisone.

Material needed:

• Sterile solution kept at −20°C (concentration: 50 μM).
• Sterile 2 mL Eppendorf tubes.

Procedure:

• Prepare aliquots of 400 μL in 2 mL tubes.
• Note on each tube the date and the content, freeze at −20°C and keep for up to 6 months.

Insulin.

Material needed:

• Sterile solution kept at −20°C (concentration: 10 mg/mL).
• Sterile 500 μL Eppendorf tubes.

Procedure:

• Prepare aliquots of 50 μL in 500 μL tubes.
• Note on each tube the date and the content, store at 4°C and keep for up to 6 months.

Epinephrine.

Material needed:

• Powder stored at 4°C.
• Sterile dd water.
• Vortex.
• Sterile 500 μL Eppendorf tubes.

Procedure:

• Dilute the powder in the sterile dd water in the concentration of 500 mg/mL.
• Prepare aliquots of 100 μL in 500 μL tubes.
• Note on each tube the date and the content, freeze at −20°C and keep for up to 6 months.

EGF.

Material needed:

• Powder (100 μg) stored at 4°C.
• Sterile dd water.
• Vortex.
• Sterile 500 μL Eppendorf tubes.
• Procedure:

Procedure:

• Dilute the powder in the sterile dd water in the concentration of 20 μg/mL.
• Prepare aliquots of 50 μL in 500 μL tubes.
• Note on each tube the date and the content, freeze at −20°C and keep for up to 6 months.

Y-27632 (ROCK inhibitor).

Material needed:

• Powder stored at −20°C.
• Sterile dd water.
• Sterile 500 μL Eppendorf tubes.

Procedure:

• Resuspend the 50 mg of the powder in 7.806 mL of dd water to reach the final concentration of 20 mM.
• Prepare the aliquots 50 μL in 500 μL tubes.
• Note on each tube the date and the content, freeze at −80°C and keep up for up to 6 months.

#### Antibiotics

Penicillin/streptomycin.

Material needed:

• 100 mL bottle of Penicillin/Streptomycin stored at −20°C.
• Sterile 15 mL tubes.

Procedure:

• Defrost the bottle in the fridge (can be done overnight).
• Divide the P/S between the 15 mL tubes in the quantity of 10 mL.
• Note on each tube the date and the content, freeze at −20°C and keep up for up to 6 months.

Fungizone (amphotericin B).

Material needed:

• 50 mL bottle of Fungizone stored at −20°C.
• 15 mL sterile tubes.

Procedure:

• Defrost the bottle in the fridge (can be done overnight).
• Divide Fungizone between the 15 mL tubes in the quantity of 10 mL.
• Note on each tube the date and the content, freeze at −20°C and keep for up to 6 months.

Gentamicin.

Material needed:

• The bottle of Gentamicin (50 mg/mL) is kept at +4°C.
• 15 mL sterile tubes.

Procedure.

• Divide between the 15 mL tubes in the quantity of 10 mL.
• Note on each tube the date and the content, freeze at −20°C and keep for up to 6 months.

Tobramycin.

Material needed:

• Bottle of Tobramycin solution is kept at room temperature (18°C–23°C).
(100 mg/2 mL of the solution, thus the concentration is 50 mg/mL).

• Sterile 500 μL Eppendorf tubes.

Procedure:

• Prepare aliquots of 200 μL in the tubes.
• Final concentration: 100 μg/mL, thus add 200 μL per 100 mL of the medium.
• Note on each tube the date and the content, freeze at −20°C and keep for up to 6 months.

Vancomycin.

Material needed:

• 1 g of vancomycin powder stored at room temperature (18°C–23°C).
• 15 mL sterile tubes.

Procedure:

• Dilute 1 g in 100 mL of dd water (concentration: 10 mg/mL).
• Divide between the 15 mL tubes in the quantity of 10 mL.
• Final Concentration: 100 μg/mL, thus add 1 mL per 100 mL of the medium.
• Note on each tube the date and the content, freeze at −20°C and keep for up to 6 months.

Meronem.

Material needed:

• 1 g of Meronem powder stored at room temperature (18°C–23°C).
• 15 mL sterile tubes.

Procedure:

• Dilute 1 g in 100 mL of dd water (concentration: 10 mg/mL).
• Divide between the 15 mL tubes in the quantity of 10 mL.
• Final Concentration: 100 μg/mL, thus add 1 mL per 100 mL of the medium.
• Note on each tube the date and the content, freeze at −20°C and keep for up to 6 months.

Colomycin.

Material needed:

• 80 mg of Colomycin powder stored at room temperature (18°C–23°C).
• 15 mL sterile tubes.

Procedure:

• Dilute 80 mg in 50 mL of dd water (concentration: 1.6 mg/mL).
• Divide between the 15 mL tubes in the quantity of 10 mL.
• Final Concentration: 100 μg/mL, thus add 1 mL per 100 mL of the medium.
• Note on each tube the date and the content, freeze at −20°C and keep for up to 6 months.

Ciprofloxacin.

Material needed:

• Sterile bags containing Ciprofloxacin solution kept at room temperature (18°C–23°C) (200 mg/100 mL).
• 15 mL sterile tubes.

Procedure:

• Divide between the 15 mL tubes in the quantity of 10 mL.
• Note on each tube the date and the content, freeze at −20°C and keep for up to 6 months.

Tazocillin (piperacillin/tazobactam).

Material needed:

• Tube of 2 g /0.25 g (or of 4 g/0.5 g) of Pip/Tazo in powder kept at room temperature (18°C–23°C) 15 mL sterile tubes.

Procedure:

• Dilute 2 g /0.25 g in 200 mL of dd water (concentration: 10 mg/1.25 mg /mL).
• Divide between the 15 mL tubes in the quantity of 10 mL.
• Note on each tube the date and the content, freeze at −20°C and keep for up to 6 months.

### Labeling of the culture flasks and plates

#### Identification

• Subject’s full name/or identification number as per local site policy.
• Type of cells (origin).
• Passage number.
• Starting date of the culture.
• Starting date of the air-liquid interface.
• Initials of the person responsible for the experiment.

#### Passage number

• At the time point when the cells are plated for the first time, passage 0 is established (P0).
• After each trypsinization, the passage number increases by one.

### Collagen coating

#### Stock solution

Material needed:

• Glacial acetic acid.
• Sterile dd water.
• 50 mg bottle of collagen type IV, Sigma-Aldrich (ref.: C-7521, stored at −20°C).
• Stirring plate.
• Syringe filter (0.45 μM).
• Sterile glass bottle.

Procedure for 100 mL of 0.2% acetic acid:

• To 100 mL of dd water, add 0.2 mL of glacial acetic acid.
• Dissolve 50 mg of collagen in 100 mL of 0.2% glacial acetic acid.
• Mix on the magnetic stirrer for at least 1 h.
• Filter sterilize the solution into the glass bottle.
• Note on each tube the date and the content; keep at +4°C for up to 6 months.

#### Working solution

Material needed:

• Stock solution.
• Sterile dd water.
• Sterile glass bottle.

Procedure:

• Dilute 1:5 the stock solution: To 80 mL of dd water, add 20 mL of collagen from the stock solution.
• Note on each tube, the date and the content; keep at 4°C for up to 6 months.

#### Coating flasks

• Use the working solution to coat the flask:
  ○ 500 μL for the 25 cm^2^.
  ○ 1 mL for 75 cm^2^.
  ○ 2 mL for 150 cm^2^.
• Use the pipette to distribute collagen evenly on the whole growing surface of the flask.
• Leave in the incubator for:
  ○ Minimum: 2 h.
  ○ Optimal: 12–16 h (overnight).
• Aspirate collagen thoroughly.
• Leave to dry in the incubator or under the hood:
  ○ Minimum: 20 min.
  ○ Optimal: 12–16 h (overnight).
• Wash with PBS:
  ○ 5 mL for the 25 cm^2^ flask.
  ○ 10 mL for 75 cm^2^ flask.
  ○ 15 mL for 150 cm^2^ flask.
• Remove the PBS.
• Let it dry in the incubator.
• Storage conditions:
  ○ Mark on each flask ‘C’ (to indicate collagen coating).
  ○ Wrap each flask with aluminum foil to protect from light.
  ○ Flasks prepared in that manner can be stored for up to 6 months at room temperature (18°C–23°C).

#### Coating filters

• Put 100 μL of the working solution on each filter.
• Leave in the incubator for at least:
  ○ Minimum: 2 h.
  ○ Optimal: 12–16 h (overnight).
• Aspirate whole collagen thoroughly.
• Leave to dry in the incubator or under the hood:
  ○ Minimum: 20 min.
  ○ Optimal: 12–16 h (overnight).
• Wash with 300 μL of PBS.
• Remove the PBS.
• Let it dry in the incubator.
• Storage conditions:
  ○ Mark on each plate with the filters: ‘C’ (to indicate collagen coating).
  ○ Seal each plate with Parafilm M.
  ○ Wrap each plate with aluminum foil to protect from light.
  ○ Filters prepared in that manner can be stored for up to 6 months at room temperature (18°C–23°C).

### Sampling with brush

Each nostril is sampled with a different brush.

**Figure 1:**
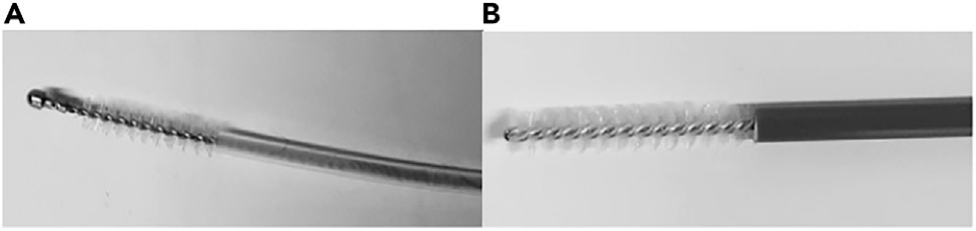
Two types of brushes for nostril sampling (A) Brush for <5 years of age. (B) Brush for >5 years of age.

For subjects under 5 years: Olympus® bronchial brush, diameter 2 mm, for 1.2 mm scope channel (see Figure 1).

For subjects over 5 years old: Gyneas® Endocervical Brush (see Figure 1).

Brushes will remain sterile if unopened.

There are therefore, per subject:

• **2 × 15 mL conical tubes containing sterile 2.5 mL of PBS**: one for each nostril.
• **2 brushes**: one for each nostril.

***Note:*** If a pediatric subject is under 5 years old, cut the wire from the brush, at approximately 20 cm from the end of the endoscopy brush and lower the sheath to release the bristles. If the child is above 5 years old, the brush does not require any alteration, as the shaft of the brush will be of adequate length for adult use.

### Materials

The following materials are required in the event that nasal brushing samples need to be sent to other research institution/s:

***Note:*** Do not forget to take solutions out of fridge 20 min before sampling.

• 2 × 15 mL tubes containing sterile 2.5 mL of FertiCult™.
• 2 × 0.5 mL tubes containing 400 μL of an antibiotic solution.

The **antibiotic solution** is composed of 100 μL of Tazocillin, 100 μL Vancomycin, Fungizone 100 μL and Colistin 100 μL. (Concentration of the antibiotic is the same as the one used for the amplification media preparation see materials and equipment: Amplification Medium).

**FertiCult™ Flushing medium** (FertiCult™, ref. FLUSH020, FertiPro NV) **-** 10% (w/v) dialyzed Ph Eurgrade polyvinylpyrrolidoneFCF (PVP) in FertiCult™ Flushing medium.

FertiCult™ Flushing medium is an aqueous solution containing physiologic salts, HEPES, lactate, pyruvate, glucose and human albumin solution.

***Note:*** We have tested cell storage in FertiCult™ for up to 48 h at ambient temperature. There was no significant loss of the cells’ viability observed.

Add 100 μL of antibiotic solution to each tube of FertiCult after sampling collection. The post-sampling addition of antibiotics is intended to avoid an allergic reaction in case the subject is sensitive to one of the antibiotics.

**Tubes with PBS (×1) or FertiCult** can be stored in the refrigerator at 4°C for 3 months.

**Tubes with antibiotic solution** can be stored in the freezer at −20°C for up to 6 months.

There is no potential hazard when dealing with the above-mentioned substances when wearing standard laboratory PPE.

## Step-by-step method details

### Sampling procedure (∼1 h)

**CRITICAL:** (Important to check before sampling) For non-cystic fibrosis participants, samples should be taken while participant does NOT have an acute infection (exacerbation).

**CRITICAL:** (Important to check before sampling) For subjects with cystic fibrosis, if a pulmonary exacerbation is in progress, the sampling should be done by adding the additional antibiotic to culture media to avoid potential contamination.

**Figure 2:**
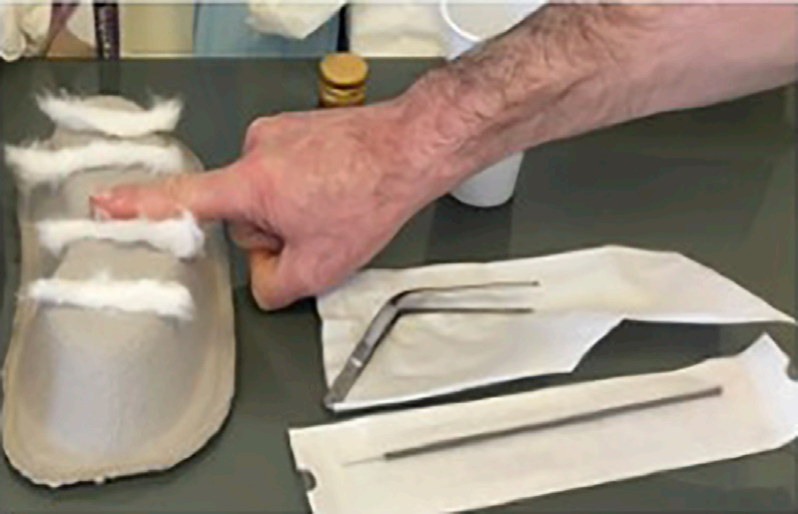
Cotton meshes, politzer tweezers, and brush for sampling procedure
  f. Ask the subject to clean/blow their nose. Ask them to sit comfortably in a chair. Explain to the subject that the procedure may cause eye watering, but this will quickly resolve.
  g. Tilt the subject’s head backwards and examine the nostrils with the speculum to:
    i. Identify the inferior turbinate (Figures 3 and 7).

1. Brush preparation and procedure (10 min).
  a. Prepare 2 × 15 mL tubes with 2.5 mL of room temperature (18°C–23°C). PBS (or FertiCult) or take pre-prepared tubes out of fridge.
  b. If using pre-prepared tubes, check the solution preparation date noted on the tube. The medium expires 3 months after the indicated date.
  c. Label the tubes with the subject’s identification/reference number and the date of sampling, put aside.
  d. Fill in the information sheet provided (see Data S1) with the subject’s identification number/tag, the genotype and if possible, information concerning the antibiotic resistance profiles of bacterial strains isolated from the subject.
  e. Prepare the sampling equipment:
    i. Sterilized nose speculum (can be sterilized with 70% ethanol or other approved sterilizing solution that is not harmful to humans).
    ii. Sterilized Politzer tweezers (tweezers are pre-sterilized; if these are not available, they can be sterilized with 70% ethanol or other approved sterilizing solution that is not harmful to humans).
    iii. Assemble 4 flat cotton meshes: these will be used to apply the local anesthetic to the nasal turbinate. Assemble two meshes approximately 5 cm and two meshes approximately 10 cm long (the longer ones will be used in the 2nd application, as they have to reach further back into the nostril area at the following point 3c). Roll the proximal end rolled into a cone, in order to introduce the mesh as far as possible up the subject’s nose (4 meshes in total per subject) (see Figure 2).

**Figure 3:**
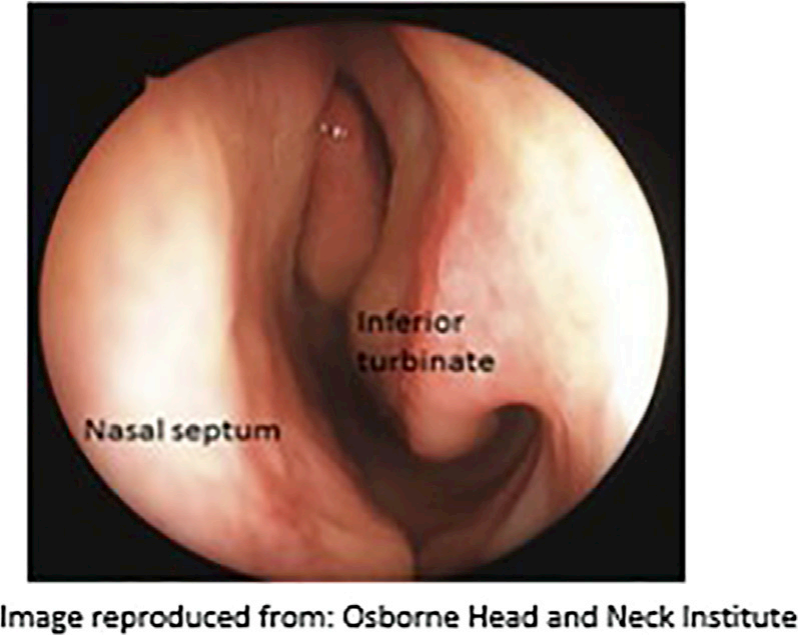
Identification of the inferior turbinate
    ii. Remove the speculum.
2. Preparation of nasal cotton meshes for administration of local anesthesia (5 min).
  a. Soak the four strands of cotton in xylocaine 5% with naphazoline solution (Figure 4).

**Figure 4:**
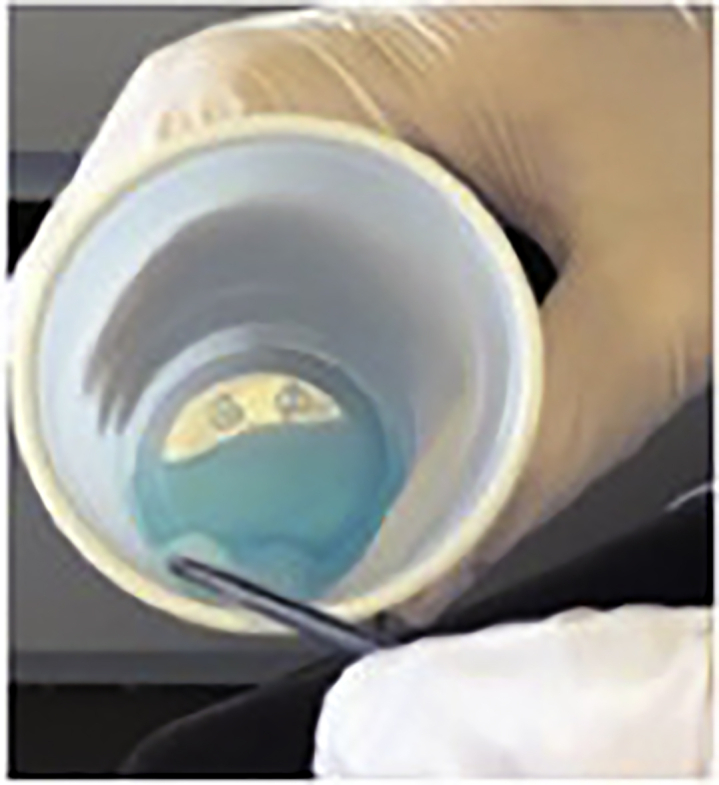
Soaking of cotton mesh in xylocaine 5% with naphazoline solution
  b. Squeeze off any excess liquid from the cotton meshes to prevent liquid dropping into the posterior pharynx to avoid unnecessary anesthesia of pharynx. Roll the end to be introduced into the nostril first (Figure 5).

**Figure 5:**
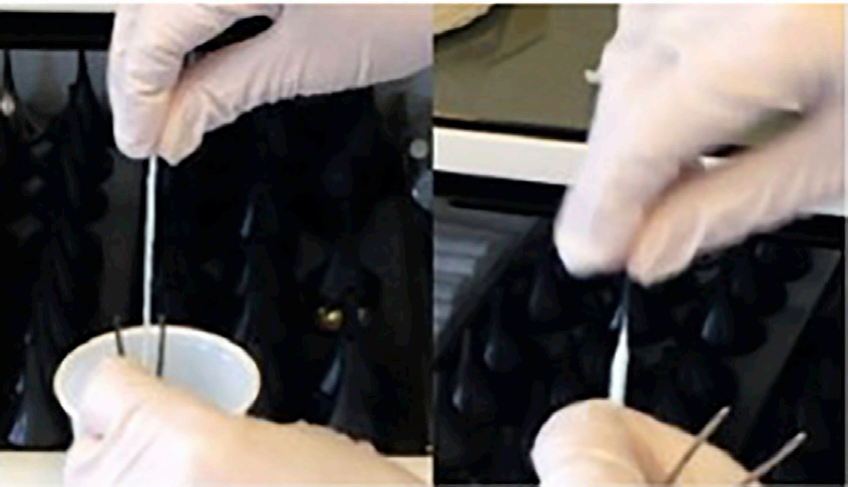
Preparation of cotton meshes for insertion into nostrils
3. Applying anesthetic (15 min):
  a. Tilt subject’s head backwards and introduce one cotton mesh (one of the 5 cm meshes) per nostril with a horizontal movement, using the Politzer tweezers. The aim is to anesthetize the proximal entry points of the nostrils (see Figure 6A).

**Figure 6:**
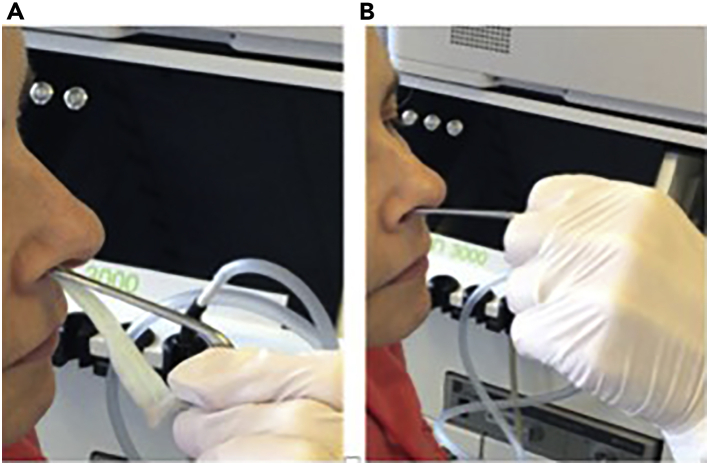
Placement of cotton mesh and nasal brush (A and B) Introducing (A) the cotton mesh into the nostril and (B) introducing the nasal brush into the nostril.
  b. Leave *in situ* for 5 min.
  c. Remove these cotton meshes and take the second set of meshes which are a little longer (approx. 10 cm), as they need to reach further back into the nostril area.
  d. Introduce one cotton mesh per nostril with a horizontal movement, using the Politzer tweezers. Take advantage of the anesthesia introduced by the first mesh insertions (now removed) to push the cotton in as far as possible.
  e. Leave *in situ* for at least 10 min.
  f. Remove cotton meshes.
  g. Ask the subject to blow their nose once more.
4. Nasal brushing technique: (10 min).
  a. Wet the nasal brush with sterile PBS (or sterile FertiCult) in a 15 mL conical tube.
  b. Introduce the nasal brush by orienting the movement horizontally i.e., having located/visualized the inferior turbinate, angle the brush horizontally along the passage between the inferior and middle turbinate, in a horizontal direction (see Figures 6B and 7 and Methods video S1).

**Figure 7:**
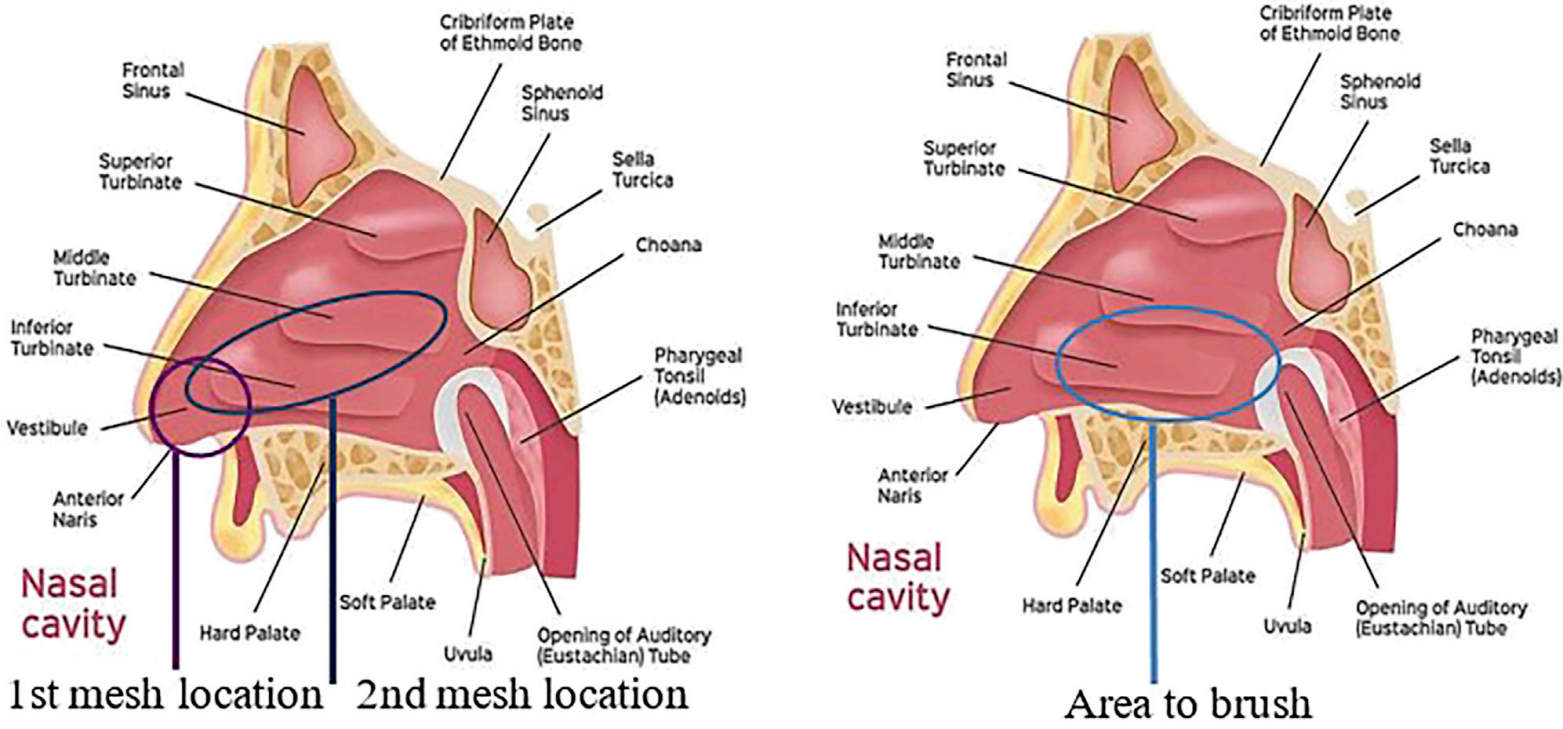
Locating the inferior turbinate: 1st and 2nd mesh localization and area to brush
    Methods Video S1. Nasal brushing technique, related to step 4
  c. The brushing should take place at the lower turbinate in the middle third (the anterior third is covered with non-ciliated epithelium).
  d. Brush with five to ten rotational and linear movements.
    ***Note:*** The subject may feel a localized tingling sensation in their nostrils and the brushing will probably cause some eye watering, but this discomfort will pass rapidly.
  e. Remove the brush from the nostril.
  f. Place the brush in tube with sterile PBS (or in the 2nd 15 mL tube with FertiCult if being sent to an alternative institution for testing) and shake the brush vigorously in the medium for 15 s.
  g. If an endoscopy brush is used, it is possible to “sweep/scrape” the brush using the plastic sheath. Normally one should see mucosal fragments floating in the solution.
  h. At a minimum, obtain one brushing per nostril. Ideally, brush each nostril twice, if possible (the same brush can be used for subsequent brushings of the same nostril).
  i. When the last brushing has been completed, shake the brush in the labeled tube and leave it inside. Cut the remaining wire if needed to allow the brush to fit entirely into the tube. Seal the labeled tube and set aside (preferably on ice or in the fridge).
  j. Repeat the procedure with the other nostril.
  k. If the tube containing FertiCult was used and the sample is meant to be sent to another center, add 0.1 mL of antibiotic solution to each of the labeled tubes containing the samples (using a sterile procedure, if possible).
  l. The labeled tubes with PBS are now ready for culture (and the labeled tubes with FertiCult for shipping). Deliver biological material to the laboratory as soon as possible, preferably on wet ice. If the brushes are kept in the Ferticult, the tubes can be transported at room temperature (18°C–23°C) within a 48-h time window.
5. Preparation of the sample before expansion: (30 min).
  After collection of the samples from the subject, the preparatory steps are as follows:
  a. Try to remove as many cells as possible from the brush. One can use the sheath from the wire, on which the brush is attached, to isolate the cells. Once cells are removed, the brush can be discarded.
  b. Centrifuge the content of the tubes at 500 × *g*, 5 min, and 4°C.
  c. Gently aspirate the supernatant avoiding touching the pellet and discard the liquid.
  d. Add 0.5 mL of Trypsin (see materials and equipment: Aliquots), resuspend the cells, and leave for 5–7 min in the incubator at 37°C.
  e. Add 1 mL of amplification medium (see materials and equipment for preparation) to stop the enzymatic process. Combine the content of the two tubes and mix gently.
  f. Centrifuge at 500 × *g* for 5 min at 4°C. Aspirate and discard the supernatant and gently resuspend the pellet in the 1 mL of amplification medium.
  g. Evenly distribute the cell suspension into collagen-coated flasks (see materials and equipment for preparation) containing:
    i. 10 mL of amplification medium (75 cm^2^ flask).
    ii. 5 mL of amplification medium (25 cm^2^ flask).
      ***Note:*** This step defines Passage 0 of the cell culture.
  h. Put the flask into the incubator at 37°C until the following day. In the most optimal conditions, it is advisable to check cells 24 h after culture initiation, allow a minimum of 12 h for the cell adhesion.
    ***Note:*** Amplification of the cells usually takes between 3–5 days after starting the culture, if the proliferation of the cells takes longer than a week, there is a risk that the cells will not grow successfully.
    ***Note:*** As a rule, a standard brush procedure (2 brushings per nostril and the 2 nostrils sampled) will allow one 75 cm^2^ flask to be seeded.
    A brushing of a single nostril can be seeded in one 25 cm^2^ flask.

**Figure 8:**
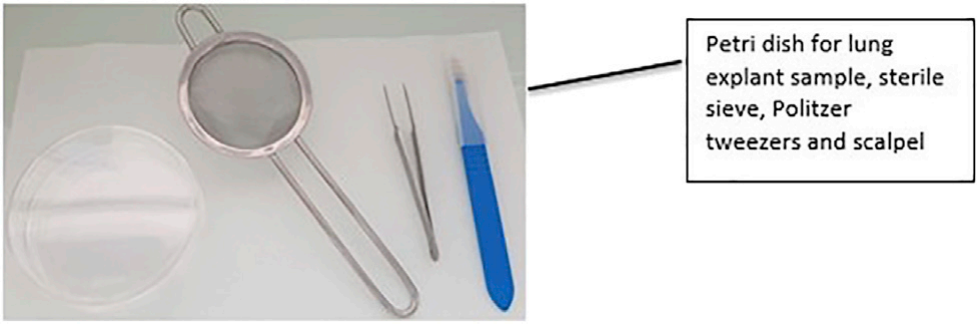
Equipment and lung explant preparation

**Figure 9:**
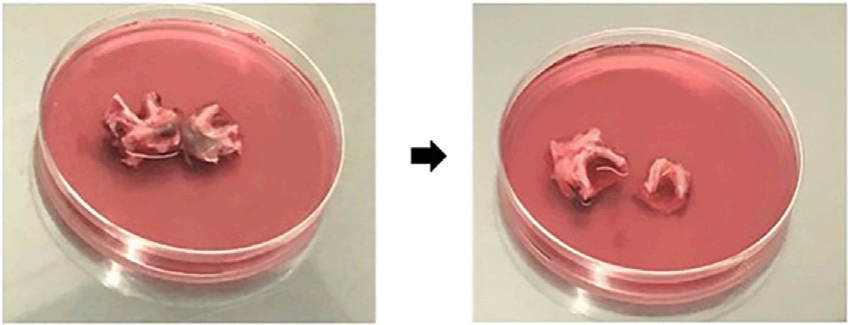
Lung explant

**Figure 10:**
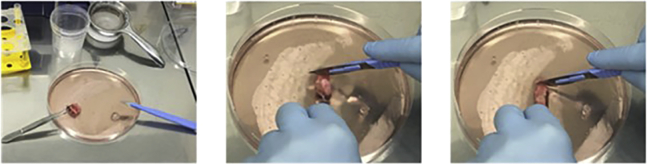
Recovery of cells from the lumen of the explant
  c. Wash the bronchi twice to remove blood cells:
    i. Add 25 mL of **PBS-DTT-FTVC** (at room temperature: 18°C–23°C) to the bronchi and shake manually for 30 s.
    ii. Aspirate media.
    iii. Repeat steps *i.* and *ii.*
      ***Note:*** DDT solution helps to remove mucus excess and lower the potential bacterial impact on contamination of the sample.
  d. Rinse the bronchi to remove the DTT:
    i. Add 25 mL of **PBS-FTVC** to the bronchi and shake for 30 s.
    ii. Aspirate media.
    iii. Repeat steps *i.* and *ii.*
      ***Note:*** The used antibiotic mix **F**: Fungizone, **T**: Tazocillin, **V**: Vancomycin and **C**: Colomycin has the purpose of preventing possible contamination of the culture with bacteria, fungi or yeast. Thus, early exposure of the human tissue to the antibiotics is of great importance.
    iv. After repeated step ii., fill the tube containing the explant with 14 mL of the pre-prepared **DMEM/F12-FTVC** medium (from the 50 mL tube, see materials and equipment: Dissociation Media), to prevent the bronchi from drying out.
  e. Dissociation of the airway epithelial cells:
    i. Add 4 mL of Pronase to the 50 mL tube containing now only 36 mL of **DMEM/F12-FTVC.**
    ii. Aspirate the 14 mL of medium from step d.
    iii. Transfer the bronchi into the **DMEM/F12-FTVC-Pronase** solution.
    iv. Incubate the bronchi over night at 4°C on a rotator (12 h for best results, possible minimal time of 4 h at 10–20 rpm - if it is possible to adjust it, as there may only be one speed set by default).
      **Pause point:** NEXT DAY (Day 2: task will take approximately 1–1.5 h).
  f. Centrifuge the 50 mL tube containing the bronchi: 500 × *g*, 5 min, and 4°C.
  g. Gently aspirate the supernatant, leaving 20 mL in the tube. Do not discard that 20 mL but pass it through the autoclaved stainless strainer at 100 μm pore size minimum to the 50 mL container/tube.
  h. Add 20 mL of DMEM/F12-FBS20%-FTVC medium to neutralize the remaining Pronase.
  i. Shake to mix well, then vortex for 2 min.
  j. Put the content of the tube (bronchi and medium) into a 15 cm Petri dish.
  k. Then bisect the lung explant with the sterile scalpel.
  l. Recover cells from the inside (lumen side) of the explant using the scalpel blade to scrape them off.
  m. Rinse the scalpel and the bronchi with DMEM/F12-FBS20%-FTVC from the petri dish to recover as many cells as possible: try to rinse the bronchi with the fast flow of the medium from the pipette.
    i. Repeat rinsing twice.
    ii. Aspirate the medium containing cells, and transfer to the 50 mL container/ tube through the stainless-steel strainer.
    iii. Rinse the Petri dish again with the remaining DMEM/F12-FBS20%-FTVC; recover the medium too, as indicated in the above point.
  n. Centrifuge the whole content of the recovered medium cell solution in the fresh 50 mL tube at 500 × *g*, 5 min, and 4°C.
    ***Note:*** Depending on the quantity of the recovered cell solution, it may need to be distributed between 2 × 50 mL tubes.
  o. Gently aspirate and discard the supernatant.
  p. Add trypsin: the amount added to the cell will depend on the pellet size as indicated in Figure 11. Between 1 to 5 mL of trypsin can be added to dislodge cells, the bigger volume of the trypsin the more carefully the incubation time should be monitored. Resuspend the cells by gently pipetting up and down, and incubate for 7 min at 37°C.

6. Lung Explant Samples (2 days: Day 1: 1–1.5 h).
  Lung explant samples (from normal or diseased lungs) are surgically supplied; it is not the remit of this manuscript to provide detailed guidance on sampling of lung explants. Briefly, bronchi after lobectomy or pneumonectomy or samples taken during lung transplantation are the sources of bronchial endothelial cells. The lung specimens are transported to the laboratory in a container filled with the PBS or culture medium, on wet ice. The lung explants should be delivered to the laboratory as soon as possible, ideally within 24 h.
  ***Note:*** Thaw the **Pronase** and prepare the medium in advance (see materials and equipment, Dissociation Media).
  Pronase is a commercially available mixture of proteases, used to help dissociate cells.
  a. After receiving the lung explant, the whole lung explant sample, including the transport media should be added (see materials and equipment: Transport medium) into the 10 cm Petri dish (all procedures to be conducted in sterile conditions).
  b. Use a scalpel blade (for this procedure, a disposable lab scalpel blade can be used with a plastic handle or as an alternative, disposable stainless-steel lancets individually foil wrapped) to remove and discard the necrotic parts of the bronchi and put the cleaned piece of the bronchi into the empty 50 mL tube (see Figures 8, 9, and 10).

**Figure 11:**
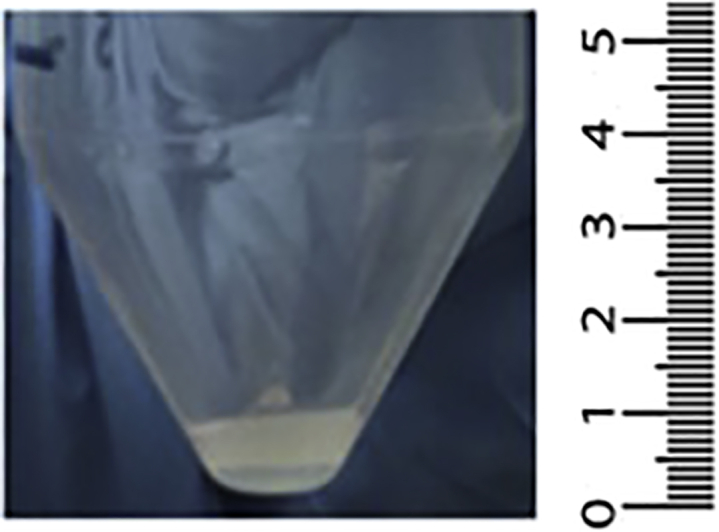
Example of the pellet to which 5 mL of medium should be added
  q. Add up to 5 mL of DMEM/F12-FBS20%-FTVC and mix the cells by gentle pipetting.
  r. Centrifuge 500 × *g*, 4°C, and 5 min.
  s. Aspirate and discard the supernatant:
    i. Add amplification media depending on the size of the pellet it will be between 2 and 5 mL and resuspend well with the pipette.
    ii. Place 15 μL of the cell suspension on a counting chamber and count the cells.
    iii. Add 10 mL amplification medium to a collagen-coated 75 cm^2^ flask (see step 9: Note, for use of the counting chamber or haemocytometer), and seed 500,000 cells per 75 cm^2^ flask.
    iv. Put the flasks into the incubator, at 37°C.
      ***Note:*This step defines passage 0 of the cultured cells (the first seeding of the cells after isolation)**.
      ***Note:*** Many of the lung specimens from diseased tissues may carry multidrug-resistant pathogens, it is therefore essential to add to the culture media specific antibiotics if the patient’s antibiogram is known.

**Figure 12:**
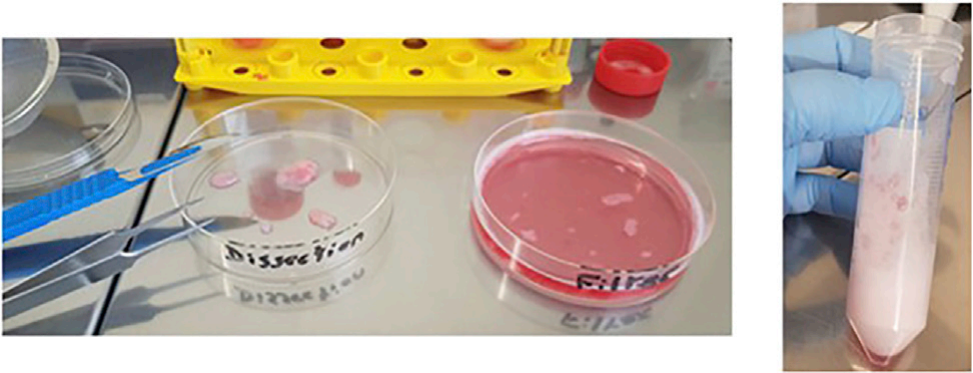
Dissociation of the airway epithelial cells from the polyp
  g. Centrifuge the tube containing the polyp at 500 × *g*, 5 min, and 4°C.
  h. Gently aspirate the supernatant, leaving 20 mL in the tube.
  i. Add 20 mL of **DMEM/F12-FBS20%-FTVC** medium to neutralize the remaining Pronase.
  j. Shake to mix well, then vortex for 2 min, at half-speed.
  k. Pass the content (polyp and medium) into a 15 cm Petri dish through a previously autoclaved stainless-steel strainer.
    ***Note:*** keep the 50 mL tube.
  l. There is no need to scratch the polyp because the cells are more easily detached than in the lung explant samples.
  m. Rinse with 5 mL of **DMEM/F12-FBS20%-FTVC**, one should try to rinse the polyp with the fast flow of the medium from the pipette:
    i. Repeat the previous step (step m) twice.
    ii. Aspirate the medium containing cells, and transfer to the initial 50 mL Falcon tube, through the stainless-steel strainer.
    iii. Rinse the Petri dish again with **DMEM/F12-FBS20%-FTVC**; recover the medium too, as indicated in step m ii above.
  n. Centrifuge the whole content at 1,500 rpm, 5 min, and 4°C.
  o. Gently aspirate the supernatant.
  p. Add between 1 to 2 mL of trypsin, (adding 2 mL if required for larger pellet size). Resuspend the cells and leave for 7 min at 37°C.
  q. Add 5 mL of **DMEM/F12-FBS20%-FTVC** and gently resuspend by pipetting.
  r. Centrifuge at 500 × *g*, 4°C, 5 min.
  s. Aspirate the supernatant.
    i. Add amplification medium (materials and equipment) (depending on the size of the pellet add between 1 and 4 mL) and resuspend the pellet with the pipette.
    ii. Place 15 μL of the cell suspension on a counting chamber and count the cells.
    iii. Put 500,000 cells per 75 cm^2^ flask, which is filled with 10 mL of **amplification medium.**
    iv. Put the flasks into the incubator until next day.

7. Polyp Samples (2 days: Day 1: 1 h).
  Polyp samples may be obtained from the surgical intervention known as a polypectomy.
  This surgical procedure can be offered to patients with isolated or syndromic nasal sinus polyposis, resistant to medical treatment and who present as symptomatic with nasal obstruction, smell disorder, chronic rhinorrhea, or repeated sinus infections.
  Specimens obtained from patients without cystic fibrosis are used as Wild Type (WT).
  The polypectomy is often associated with the opening of the sinuses. It is an endoscopic intervention, that is, through the nasal cavity with a camera. It is performed under general anesthesia. The patient leaves the evening or the day after the operation. The polyps are then transported in sterile PBS or culture medium in the 50 mL tube on wet ice.
  a. Thaw the Pronase and prepare the dissociation media in advance (see materials and equipment).
  b. Aspirate transport medium (be careful to not aspirate small polyps).
  c. Place the polyp in an empty 50 mL falcon tube.
  d. Wash the polyp to eliminate blood cells:
    i. Add 25 mL of **PBS-DTT** to the polyp and shake manually for 1 min.
    ii. Aspirate medium.
    iii. Repeat the steps *i.* and *ii.*
      ***Note:*** DDT solution helps to remove excess mucus and lower the potential bacterial impact on contamination of the sample.
  e. Rinse the polyp to remove the DTT:
    i. Add 25 mL of **PBS-FTVC** to the polyp and shake for 1 min.
    ii. Aspirate medium.
    iii. Repeat the steps *i.* and *ii.*
      ***Note:*** The used antibiotic mix **F**: Fungizone, **T**: Tazocillin, **V**: Vancomycin and **C**: Colomycin has the purpose of preventing possible contamination of the culture with bacteria, fungi or yeast. Thus, starting the exposure of the human tissue to the antibiotics at the earliest stage is of great importance.
    iv. From the 50 mL tube with the ready medium **DMEM/F12-FTVC** take 14 mL and add it to polyp, it will prevent the polyp from drying out.
  f. Dissociation of the airway epithelial cells (see Figure 12):
    i. In the 50 mL tube containing 36 mL of **DMEM/F12-FTVC** add 4 mL of Pronase.
    ii. Aspirate the 14 mL of medium (from step e: iv. above).
    iii. Transfer the polyp into the **DMEM/F12-FTVC-Pronase** solution.
    iv. Incubate the polyp overnight (12 h minimum) at 4°C on a rotator, at 10–20 rpm if it is possible to adjust it, as sometimes there is only one speed set by default.
    v. Note the time when the polyp is placed in the Pronase solution.
      **Pause point:** NEXT DAY (Day 2: 1 h).

This step defines passage 0 of the cultured cells (the first seeding of cells after isolation).

***Note:*** Do not forget that antibiotics are being adjusted according to the subject’s antibiogram, if no information is available; add antibiotics indicated as a ‘standard’ in the protocol.

8. Expansion (3–5 days).
  From the expansion stage in the flasks, the following steps are common regardless of the initial origin of the cell (brushing/explant/polyp).
  a. The day after seeding and only on that day: (Depending on number of flasks it will take from 30 min to a few hours).
    i. Aspirate medium from the 75 cm^2^ or 25 cm^2^ flask.
    ii. Rinse with 10 mL of PBS-FTVC.
    iii. Aspirate PBS-FTVC.
    iv. Add fresh 10 mL (for a T75 flask) or 5 mL (for a T25 flask) of **amplification medium.**
  b. Inspect the cells every day for:
    i. Adherence (clumps of cells that did not attach to the culture vessel).
    ii. Shape (the epithelial cells are columnar and non-ciliated, when stretched on a culture dish: with large cytoplasm and one nucleus) (see Figure 13).

**Figure 13:**
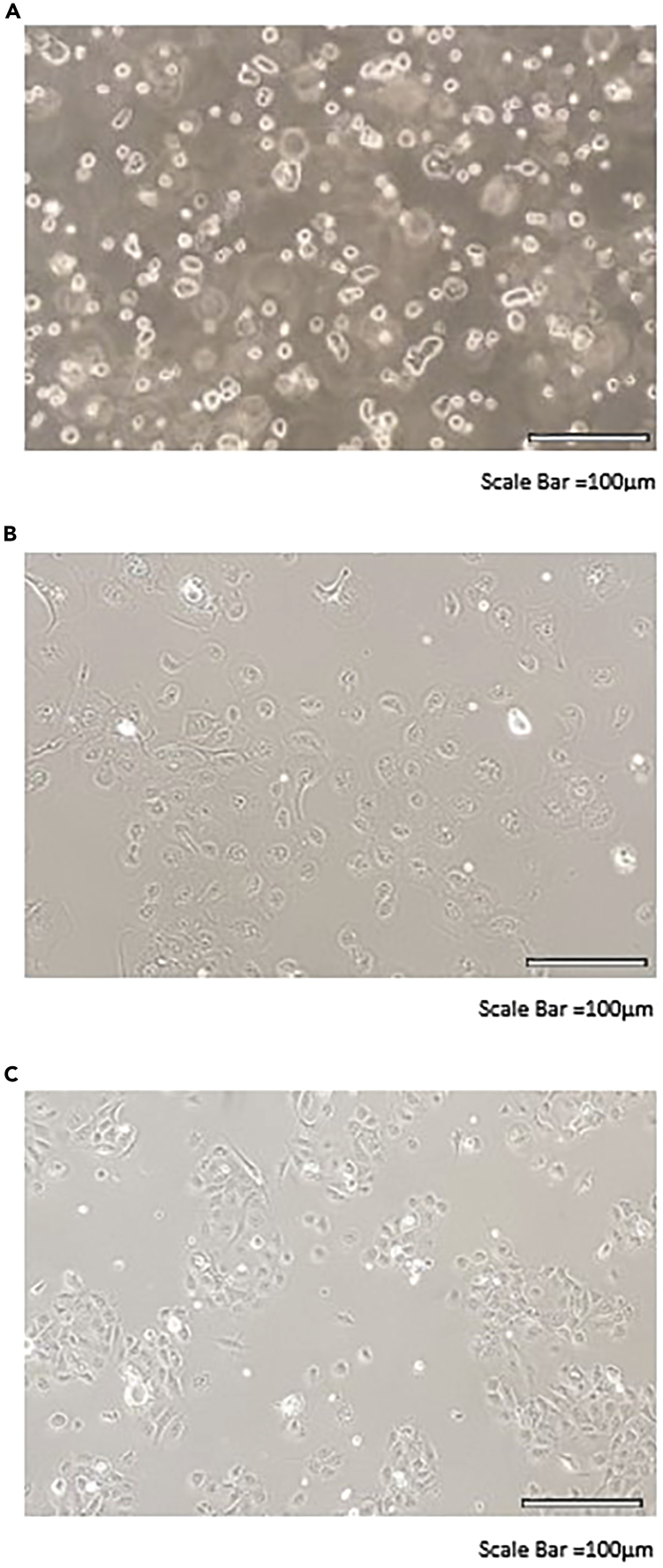
Cell expansion (A) Cell suspension. (B) Cells after 1 day in culture. (C) Cells after 3 days in culture (60% of confluence).
    iii. Growth (cells should slowly increase in quantity and grow across the plastic surface).
    iv. Quality of the medium (it should be clear of pieces of cells or their floating aggregates).
  c. Change the medium every 48–72 h: standard procedure.
    i. Aspirate medium.
    ii. Add fresh 10 mL (for a T75 flask) or 5 mL (for a T25 flask) of **amplification medium.**
      ***Note:*** The expansion can take from 3 to 5 days depending on cell number (See Figure 13C).
      The cells are ready to be passaged when they reach 80%–90% of confluence (only small holes in the cells monolayer are visible). If it the expansion of the cells takes longer, i.e., more than 10 days, the culture might not be successful.
  d. If medium is not clear (i.e., cloudy), this could indicate a bacterial or yeast infection in the medium, in which case:
    i. Sample the medium and send the aliquots to the microbiology department for bacterial analysis (to assess if bacterial contamination is from the patient or from laboratory contamination).
    ii. Rinse the cells in the flask with 10 mL of adapted PBS-FTVC.
    iii. Add 10 mL of amplification medium with antibiotics indicated in the antibiogram (if available).
    iv. In the event of an absence of antibiotic information specific for the patient, add Ciprofloxacin (materials and equipment).
      ***Note:*** Avoid aminoglycoside antibiotics, especially for cells cultured from subjects with codon stop mutations in the CFTR gene.
    v. Notify the lab manager about possible contamination of medium and incubator and follow laboratory procedures for disinfection and isolation of exposed flasks.
  e. Freezing.
    i. A portion of the expanded cells after the first passage should be frozen for bio banking; ideally, 0.5–1.0 × 10^6^ cells should be frozen per cryovial (according to the following step 13).
    ii. In case of an insufficient number of cells, freezing can be performed with the cells from passage 1 (P1).
    iii. Regarding rare and interesting genotypes, the cells should be frozen also at P1 and P2.
  f. General guidelines:
    i. Never seed cells directly onto filters after sampling: the expansion phase is needed.
    ii. In the incubator, try to separate WT and CF cultures, for example placing the clearly labeled WT cultures on a separate, clearly labeled shelf.
    iii. If contamination is suspected in some flasks, change medium in these flasks at the end of cell culture handling.
9. **Seeding cells onto filters:** (Depending on number of flasks from 1 h to few hours).
  First, discuss the number of filters needed, with members of the laboratory staff: unseeded cells will be further propagated if the passage of the expansion is lower than P2 or frozen.
  a. Pre-coat filters with collagen as shown in materials and equipment.
  b. Aspirate and discard culture medium from 75 cm^2^ culture flask/s.
  c. Add 10 mL of PBS to cell culture flask.
  d. Aspirate and discard PBS.
  e. Add 2 mL of **Trypsin** (see materials and equipment) for a 75 cm^2^ flask, and leave for 7 min at 37°C.
  f. Tap the flask firmly, with the palm of the hand, to help the cells detach. One should keep the flask in a horizontal position to avoid spreading the suspension.
  g. Add 10 mL of amplification medium to stop the enzymatic reaction. Vigorously rinse the flask, using the 10 mL pipette to draw up and expel the amplification medium over the flask surface to rinse and detach cells.
    ***Note:*** Check the trypsinized flask under the microscope. If there are still many cells attached to the flask surface, repeat the trypsinization, by adding 2 mL trypsin and incubating at 37°C for 5 min maximum.
  h. Transfer the cell suspension to a 50 mL tube and centrifuge at 500 × *g*, 5 min, and 4°C.
  i. Aspirate and discard the supernatant.
  j. Add 3–5 mL of amplification medium depending on the size of the pellet, resuspend pellet with the 10 mL pipette.
  k. Take 15 μL of the cell suspension, place on a cell-counting chamber, count and calculate cell density of suspension.
    ***Note:*** In this SOP, the Malassez counting chamber is used. Different counting chambers may be used in different laboratories such as Bürker Türk, Neubauer-improved or haemocytometer but the spacing of the grid and volume of the chamber must be known.
    ***Note:*** Count the number of cells in 10 small squares (as shown in Figure 14) (Stokes et al., 2014).
    **Figure 14:**
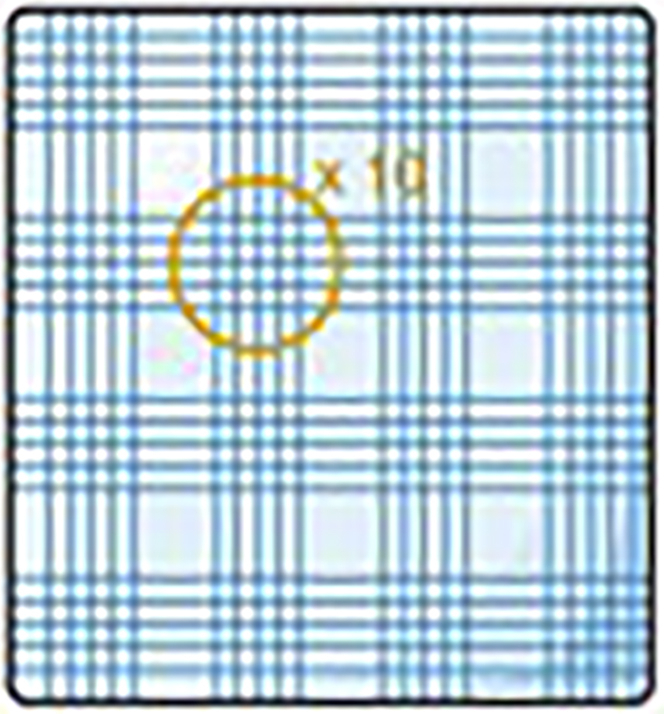
Counting cells
  l. To calculate the previously defined volume on the apical side of the filter follow the technical counting note as follows:
    i. Total number of cells**=** number of counted cells **×** 10^4^ **×** volume in mL of the medium in which the cells were suspended in the tube.
    ii. Number of filters feasible to prepare**=** Total number of cells **÷** Number of cells per filter.
    iii. Minimum 330,000 cells for a filter size of 0.33 cm^2^, up to 350,000 cells / filter.
    iv. Volume of the cell suspension to be put on apical side of the filter**=** volume in mL of the medium in which the cells were suspended in the tube (quantity used in step j above) **÷** Number of filter feasible to prepare.
  m. The maximal apical volume is 300 μL, if the calculated volume is smaller than that; one should complete it up to 300 μL with amplification medium (before adding the cells).
  n. The volume of basal medium is 900 μL.

Prepare a 24-well plate with the desired number of collagen-coated filters.

10. Maintenance of filters before air-liquid (ALI) interface.
  (Depending on number of filter plates from 30 min to few hours).
  a. The apical medium is not removed before day 2–3. If the apical medium is left for longer (up to 5 days until the cells fill up the filter area), exchange it for fresh 300 μL of the amplification medium.
  b. The basal medium is changed every 48–72 h. Aspirate and discard basal medium and add 900 μL of fresh ALI medium.
    i. If medium is not clear (i.e., cloudy), and there is suspicion of contamination, collect the medium and send it to the bacteriology department for testing.
    ii. If the apical medium appears cloudy before starting ALI culture:
      Aspirate and discard the apical medium.
      Rinse with 300 μL of adapted PBS-**FTVC**.
      Add fresh 300 μL of amplification medium with change of antibiotics according to the antibiogram.
  c. ALI can be initiated 2–5 days after cells are seeded onto the filters.
  d. From now on, always use different pipette tips for changing the medium on the apical and basal sides of the filter (to avoid cross-contamination).

11. Initiation of ALI phase (change basal medium every 48–72 h).
  a. Aspirate and discard the apical medium. From now onwards, the apical side remains medium-free.
  b. Aspirate and discard basal medium.
  c. Add 900 μL of fresh ALI medium in basal part of well.
  d. Change the basal medium every 48–72 h.
  e. Cell differentiation will now take between 15 to 30 days (from start of air-liquid phase) to the point of mature epithelium.

***Note:*** Poor quality of the filter is suspected if the ALI medium starts to pass over onto the apical side of the filter (this may happen around 7 days after launching the ALI culture).

After about 2 weeks at the ALI, cells start to differentiate, and cilia appear.

It is very important to remove CIPROFLOXACIN and AMINOGLYCOSIDE ANTIBIOTICS from the culture medium 5 days before planned.

**Figure 15:**
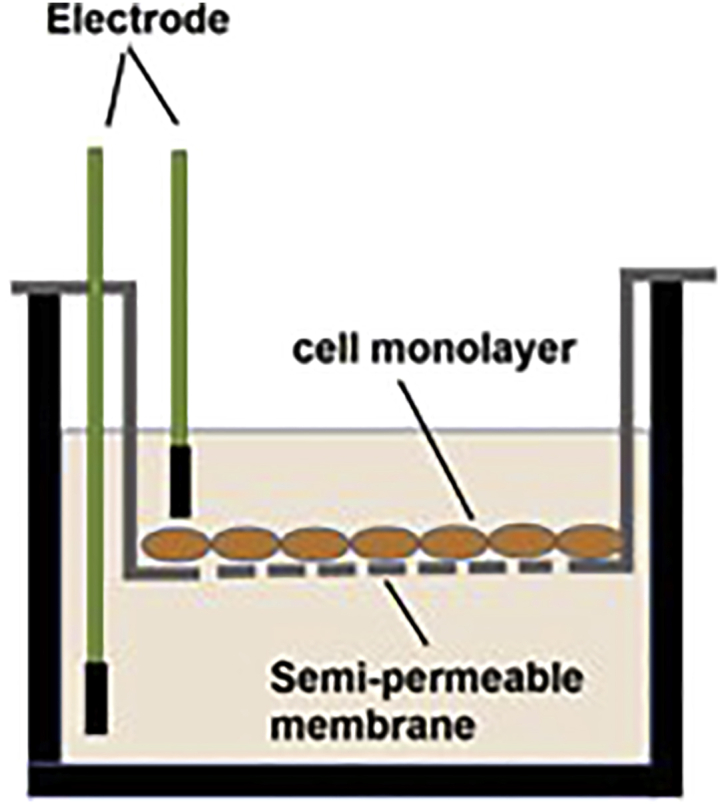
Measurement of the transepithelial electrical resistance (TEER)
  For resistance measurements (according to the manufacturer manual):
  a. Turn the **Power** on, on the **EVOM2.**
  b. Sterilize the electrode with 70% ethanol (submerge the electrodes for several seconds) and air dry.
  c. Connect the electrode to the meter.
  d. Set the **Function** switch to **Ohms.**
  e. Turn the Mode Switch to **R.**
  f. Turn the range switch to **20k** (kΩ, ×1000 = Ω).
  g. Measure the blank resistance and record the value (R blank).
    i. The short electrode should be placed in the insert (apical compartment), the longer arm of the electrode is located in the basolateral compartment.
    ii. All movement should be performed gently to avoid damage of the cell monolayer.
    iii. Push the measure R button.
    iv. Record the given number which will be expressed in kΩ.
  h. Measure the sample (R sample) in the same manner. It is advisable to perform the measurements in duplicates.
  i. Perform the measurements in rather fast pace as the temperature may impact the results (one method to minimize temperature-induced variability in TEER measurements between samples is to place the culture plate on a pre-warmed 37°C heat block during measurements).
  j. To obtain the reading: (R (sample) Ω - R (blank) Ω) **×** 0.33 cm^2^ (membrane area).
  k. Clean, dry and store the electrode.
    ***Note:*** If evaluation of epithelia by TEER is conducted when the cells are already under ALI conditions, sterile physiological saline solution should be added on the apical side to allow electrical contact with the electrode. A volume of solution sufficient to allow insertion of the apical electrode should be used (∼100 μL). After measurement, the saline solution must be removed to return to ALI condition.

12. Measurement of the transepithelial electrical resistance (TEER) (30 min).
  This is recommended to prepare the filters for use in different assays. This method allows verification of the integrity of the tight junctions in the monolayer of epithelial cells and is carried out without damage to the cells (Srinivasan et al., 2015). (The EVOM2 can be used, the most widely used manual epithelial volt/ohm meter). Whilst this procedure is not the remit of this protocol, a brief outline is provided below. For more detailed procedural instructions, please refer to the manufacturer’s instructions.
  (See Figure 15).

For further guidance, please refer to: https://www.wpiinc.com/media/wysiwyg/pdf/EVOM2_IM.pdf (the meter models may differ).

13. **Freezing cells** (Depending on number of flasks from 30 min to 1.5 h).
  a. Prepare freezing medium (see materials and equipment).
  b. Prepare cryotubes with the name of the subject/identification number (as per local site policy), site ID, the type of cells, passage number, date of freezing, initials of the person performing the freezing and if possible, the number of frozen cells.
  c. Freeze cells at P0 and at no higher than P2.
  d. Choose a 75 cm^2^ flask of cells (P0-P2) at 80%–90% confluence.
  e. Aspirate and discard the medium from the flask.
  f. Add 10 mL of PBS.
  g. Remove the PBS (the purpose of steps f and g is to remove residual medium from the flask).
  h. Add 2 mL of trypsin and put into incubator for 7 min.
  i. Tap the flask, with the palm of your hand, to help the cells detach. One should keep the flask in a horizontal position to avoid spreading the suspension.
  j. To stop the trypsin activity, add 10 mL of amplification medium.
  k. Rinse the cell-growing surface well.
  l. Repeat the process several times trying to produce a strong flow in order to detach as many cells as possible.
  m. Transfer the cell suspension to a 50 mL tube.
  n. Centrifuge at 500 × *g*, 4°C, and 5 min.
  o. Remove the supernatant. Resuspend the cell pellet in a sufficient (refer to the pellet size) amount of the regular medium. Count the cells and spin the whole content of the tube.
  p. In each cryovial, freeze around 1–2 × 10^6^ cells per 1 mL of freezing medium.
  q. Resuspend the cells in the desired amount of freezing medium. Re-distribute equal amounts of cell suspension to each cryotube.
  r. Place the cryovials in Mr. Frosty (Nalgene) and put into a −80°C freezer.
  s. The next day, move the preserved sample to the nitrogen storage container, where the cells can be kept for many years.
14. Defrosting (30 min).
  a. Prepare the materials needed:
    i. Warm up the water bath to 37°C.
    ii. Prepare the amplification medium.
    iii. Warm up the medium to 37°C.
    iv. 15 mL tube(s) and 75 cm^2^ flask(s).
  b. Remove cryovials from the nitrogen storage tank.
  c. Leave the cryotube for a few minutes in the water bath (up to 2 min), taking care not to submerge the whole vial in the water.
  d. Note the label on the vial before wiping it with alcohol, to avoid losing the information from the cryotube.
  e. Using a 1 mL pipette, transfer the defrosted cells to the empty 15 mL tube.
  f. In a drop-wise manner, add 1 mL of the warm amplification medium.
  g. After one minute, again add 1 mL of amplification medium and wait an additional minute.
  h. Next fill the tube with 10 mL of amplification medium and centrifuge for 2 min at 230 × *g*.
  i. Aspirate and discard the supernatant.
  j. Check what number of frozen cells is indicated on the cryovial. Resuspend the cell pellet in the volume of amplification medium required to achieve, at least, a cell density of 1 × 10^6^ cells/mL.
  k. Put the cell suspension into the flask(s) containing a sufficient amount of the medium, as follows:
    i. 75 cm^2^ flask: seed up to 2 million cells to a total volume of 10 mL amplification medium.
    ii. 150 cm^2^ flask, seed up to 4 million cells to a total volume of 15 mL amplification medium.
  l. Place the flasks in the 37°C incubator and observe cell expansion over the course of the next 2–3 days.

END OF PROCEDURE.

## Expected outcomes

### Cell expansion

This can take from 3 to 5 days depending on cell number (See previous Figure 13C).

The cells are ready to be passaged when they reach 80%–90% of confluence (only small holes in the cells monolayer are visible). If it the expansion of the cells takes longer, i.e., more than 10 days, the culture might not be successful (See Figure 13).

### Suspension filter

After about 2 weeks at the ALI, cells start to differentiate, and cilia appear.

**Figure 16:**
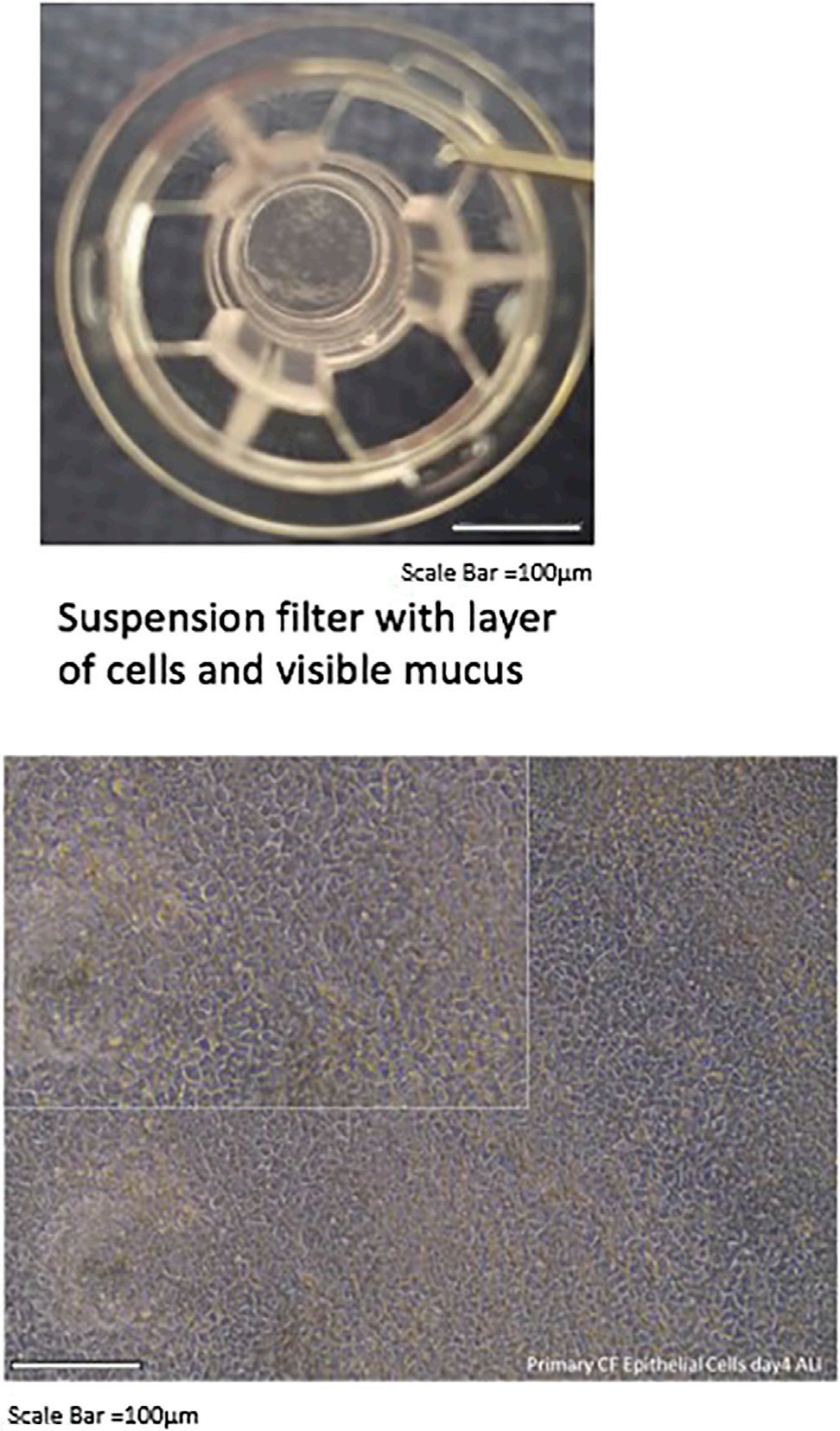
Suspension filter with layer of cells and visible mucus and layer of epithelium after 10 days in the air-liquid culture

**Figure 17:**
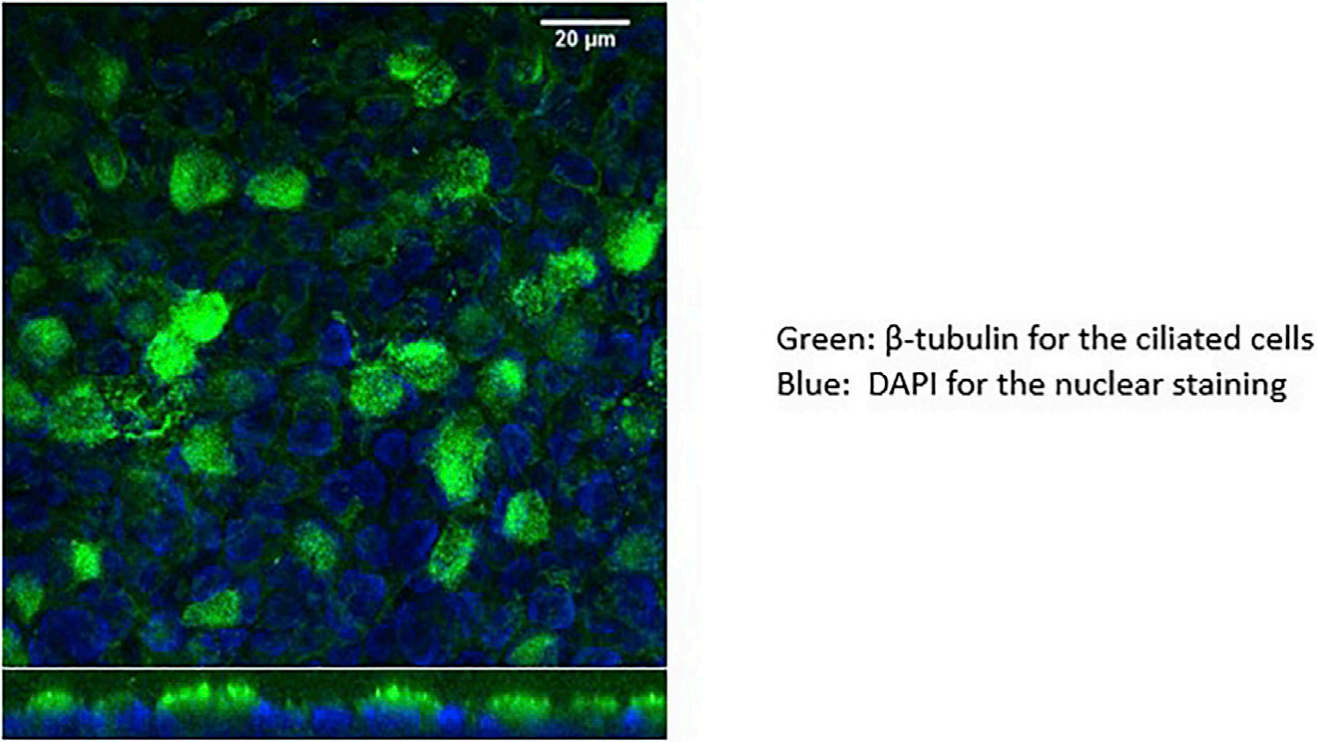
Ciliated cells after approximately 2 weeks at the ALI

See Figure 16 and Figure 17.

## Limitations

Possible limitations of this protocol may include the following:

There may be variation from laboratory to laboratory in terms of the use of antibiotics. This protocol represents the authors’ preferred methods of choice and any variation in local site procedure should be discussed on a case-by-case basis.

Similarly, there may be site-to-site variation in terms of use of different media for amplification.

For example, use of a “home-made” serum-free medium.

Sites should adhere to this protocol as able and any variation from this protocol should be discussed on an individual site basis and carefully documented/recorded.

Likewise, checks for possible SARS-CoV-2 infection before (patient tests positive) or after (in the fluid) brushing, should be agreed in line with local infection control procedures and are not the remit of this protocol.

## Troubleshooting

### Problem 1

When cells are not growing well and are very sparsely seeded (step 8).

### Potential solution

The FBS concentration can be increased by 5%–10% for few days, if this boost will not trigger the amplification of the cells, it may mean that the culture is unsuccessful and needs to be discarded.

### Problem 2

The cell layer on the filters in ALI after few days of culture seems patchy/uneven and the basal medium starts to appear in the apical compartment (step 8).

### Potential solution

It may mean that the seeded amount of the cells was not sufficient or the apical medium was withdrawn too fast. Do not use these filters further.

### Problem 3

The filters could not be clamped in the Ussing experiment (steps 9–11).

### Potential solution

It is recommended to measure the TEER which should show a value of over 200 Ω∗cm^2^, before application of the filters in the Ussing chamber.

## Resource availability

### Lead contact

Further information and requests for resources and reagents should be directed to the lead contact: Anita Golec (anita.golec@inserm.fr).

### Materials availability

This study did not generate new unique reagents.

## Data Availability

This study did not generate/analyze data sets/codes.
