# Supplementary material for: Isolation, cultivation, and application of primary respiratory epithelial cells obtained by nasal brushing, polyp samples, or lung explants
Source: STAR Protoc. 2022 May 27;3(2):101419. doi: 10.1016/j.xpro.2022.101419 (PMC9157560; doi:10.1016/j.xpro.2022.101419)
Supplement: Document S1. Data S1 [file mmc1.pdf]

APPENDIX 8: Information Sheet: reference to Step 1d in the protocol:

Isolation, cultivation and application of primary respiratory epithelial cells obtained by nasal brushing, polyp samples or lung explants.

Information Sheet:

PATIENT'S TAG/ID:..
Family name/surname: ....
First name: ....
Sex: F / M
Date of birth: ....
Patient's GENOTYPE: ....
Please state: circle as appropriate

PSEUDOMONAS AERUGINOSA:	Yes	No
CIFLOX	Yes	No
CEFTAZIDIME	Yes	No
TAZOCILLIN	Yes	No
COLI	Yes	No
STAPHYLOCOQUS AUREUS	Yes	No
AMOX AC CLAV	Yes	No
TICARCILLIN	Yes	No
VANCOMYCIN	Yes	No
ECHERICHIA COLI:	Yes	No
FUNGI:	Yes	No

Doctor on duty: ....
Name of the person who performed the brushing: ....
Date and time of the brushing: ....

Additional notes:
Please note any sinonasal symptoms: ....
Inflammatory mucus on inspection:...
Date and details of the last doses of antibiotics taken:
....
....
....
IF POSSIBLE STATE THE LAST ANTIBIOTIC RESISTANCE PROFILES OF BACTERIAL STRAINS ISOLATED FROM THE PATIENT:
....
....
